# Improved Method of Circumcision

**Published:** 1883-10

**Authors:** 


### IMPROVED METHOD OF CIRCUMCISION.

Neil Macleod suggests a change in the method of performing circumcision. He dilates the orifice in the prepuce with forceps, making also, if necessary, very slight snips with scissors around the margin of the dilated orifice, until it will admit of retraction over the glans. Adhesions are broken down by means of a probe passed between the glans and the prepuce. The whole glans having been exposed, the prepuce is then drawn forward again, and a clip, formed by tying together two ordinary directors, is slipped over the loose end of the prepuce, marking the amount to be cut off. Three carbolized silk threads are then passed, at equal intervals, through the prepuce close to the clip on the proximal side, the glans being protected as the needle was passed, and the threads long enough each for two sutures. The prepuce in front of the clip is then cut off close, the clip removed, vessels twisted or tied if preferred; the threads are fished up with a blunt hook from the now enlarged preputial slit, cut and tied on each side. The orifice in the mucous layer may then be slit up to the corona, if necessary; but if the clip be so placed as to run in the direction from the meatus to the corona, this will be unnecessary. Any simple dressing may be used.—*Edin. Med. Jour.*, March, 1883.

